# Comparing the Relative Importance of Water-Borne Cues and Direct Grazing for the Induction of Defenses in the Brown Seaweed *Fucus vesiculosus*

**DOI:** 10.1371/journal.pone.0109247

**Published:** 2014-10-03

**Authors:** Carla R. Flöthe, Uwe John, Markus Molis

**Affiliations:** 1 Section Ecological Chemistry, Alfred-Wegener-Institut, Helmholtz-Zentrum für Polar- und Meeresforschung, Bremerhaven, Germany; 2 Section Functional Ecology, Alfred-Wegener-Institut, Helmholtz-Zentrum für Polar- und Meeresforschung, Bremerhaven, Germany; University of New South Wales, Australia

## Abstract

Some seaweed species have been shown to release water-borne cues after herbivore attack, for example, to attract natural enemies of the herbivore. These cues may also be sensed by neighboring seaweeds and used to adjust their defenses in anticipation of a possible herbivore attack. Several studies indicated information transfer between seaweed individuals in the past, including the brown seaweed *Fucus vesiculosus*. Previous work showed induction of defenses in *F. vesiculosus* in response to water-borne cues released by isopod-grazed conspecifics. In contrast, another study on induced responses after exposure to cues from isopod-grazed neighbors using the same seaweed species yielded contradictory results. This study reassessed the ability of *F. vesiculosus* individuals to sense water-borne cues released by isopod-grazed neighbors in a series of experiments that monitored *F. vesiculosus* palatability in response to direct grazing by *Idotea baltica* and water-borne cues from isopod-grazed neighbors relative to unmanipulated seaweed pieces. Two-choice feeding assays were conducted with both fresh and reconstituted seaweed pieces. Direct grazing by *I. baltica* induced a chemical defense in *F. vesiculosus*, confirming results of previous studies. In contrast, evidence for increased herbivore resistance in seaweed pieces that were located downstream of isopod-grazed *F. vesiculosus* could not be provided. The lack of defense induction in response to grazing of conspecific neighbors may be explained by the environmental conditions and the scattered distribution of *F. vesiculosus* individuals in the intertidal zone of Helgoland, which may render resource investment in the emission and/or response to water-borne cues at this site unprofitable.

## Introduction

The ability of seaweeds to induce defenses in response to attack by herbivores is well known and a meta-analysis revealed that brown and green seaweeds frequently induce chemical anti-herbivory defenses [1]. The induction of defenses is suggested to be associated with the release of info-chemicals in some seaweed species, which may act as an indirect defense mechanism by attracting and directly influencing the foraging behavior of natural enemies of the attacking herbivore [2]. As the induced anti-herbivory defense does not necessarily spread to adjacent undamaged thallus parts [3] and almost all seaweeds have a limited internal transport system, these info-chemicals may also optimize within-plant signaling to ensure a systemic defense without receiving damage at all thallus parts [4].

At the same time, emitted info-chemicals become ‘public’ information and may also be sensed by other seaweeds. Three decades ago, researchers discovered that vascular plants apparently ‘listen’ to their neighbors, since herbivore attack resulted in increased resistance to herbivory not only in the attacked plant but also in plants growing nearby [5]. Thus, ‘communication’ between herbivore-damaged and undamaged neighbors may allow induction or preparation of defenses prior to attack [6]. Toth and Pavia [4] initially demonstrated that the brown seaweed *Ascophyllum nodosum* sensed water-borne cues released by periwinkle-grazed conspecific neighbors and increased secondary metabolite production without being subjected to direct grazing. Further studies reported that water-borne cues induced defenses in several green, red and various brown seaweed species [7]–[13]. Other studies failed to detect induction of anti-herbivory traits after exposure to info-chemicals (e.g. [14]) and ‘communication’ between adjacent seaweeds was suggested to be dependent on both the attacked seaweed species [8] and the attacking herbivore species [10]. However, the detection of defenses induced by water-borne cues may be complicated by highly variable temporal defense patterns. *Ascophyllum nodosum*, for example, was shown to use an oscillating temporal defense pattern in response to water-borne cues from grazed neighbors [15]. Although info-chemical triggered anti-herbivory traits may have been overlooked in some macroalgae due to temporally variable defenses, ‘communication’ via water-borne cues may indeed not occur in all seaweed species for several reasons. First, emitters of info-chemicals may be faced with metabolic costs in terms of production, transport, storage and release (e.g. [16]) and maintenance costs may arise for the synthesis of enzymes involved in these processes (e.g. [17]). Thereby, water-borne cues may incur particularly high costs because they are released into the environment and may have to be renewed constantly. Second, once water-borne cues are released, they may not only attract the herbivore's predator, but also other members of the food web, such as other herbivore species or ineffective, but competitively superior natural enemies (e.g. [17], [18]). Third, neighboring seaweeds are likely to compete for resources [19]. Helping adjacent seaweeds may shift the competitive balance between emitter and receiver towards a disadvantage for the emitter, which already received damage but possibly helps its competitor to successfully escape herbivore attack.

In this study, induced anti-herbivory traits were investigated in *Fucus vesiculosus*, a common brown seaweed in the intertidal zone of North Sea shores [20]. *Fucus vesiculosus* was shown to sense cues from conspecific neighbors grazed by the isopod *Idotea baltica* in some studies [10], [13], while Yun and colleagues [8] reported contradictory results about the ability of *F. vesiculosus* individuals to ‘communicate’ with each other. They showed that the exposure to water-borne cues from *I. baltica*-grazed neighbors reduced the palatability of reconstituted *F. vesiculosus* pieces, but not that of live seaweed pieces. As *F. vesiculosus* was shown to use an oscillating temporal defense pattern in response to direct grazing by isopods and periwinkles [21], [22] and seaweed defenses triggered by water-borne cues may also show temporal variability [15], this inconsistency may have been caused by a temporal mismatch between feeding assays and single defense ‘pulses’ of water-borne cue-exposed *F. vesiculosus*.

A series of laboratory experiments was used in this study to (1) reassess whether *F. vesiculosus* responds to water-borne cues from conspecific neighbors grazed by the isopod *I. baltica* and (2) initially test for temporal variation in the efficacy of water-borne cue-exposed *F. vesiculosus* to deter grazers.

## Materials and Methods

Organisms used in this study were collected during low tide in the mid rocky intertidal at Kringel, Helgoland, NE Atlantic (54°10′60″N, 7°53′15″E) in July 2012. At this site, the intertidal is dominated by perennial canopy-forming brown seaweeds, such as toothed wrack (*Fucus serratus*) and bladder wrack (*F. vesiculosus*), as well as irish moss *Mastocarpus stellatus*. The isopod *I. baltica* is a littoral mesograzer species [23], but can also be found among drift algae of the genera *Fucus* and *Ascophyllum* [24]. All isopods used for this study were taken from an *I. baltica* culture that was fed with customary fish food and *Ascophyllum nodosum*, and maintained in an aerated 200 L flow-through tank with a 12/12 h light/dark cycle within a constant temperature room at 15°C. Every year, new individuals from drift algae collected in the Helgoland Bight were introduced into the culture.

This study was conducted in compliance with the legal requirements of the state order no. 791-4-37 issued by the government of the Land Schleswig-Holstein on 24 April 1981 that declared the rocky shores below the high tide limit of Helgoland Island a nature reserve and allows ecologists to access sites to accomplish field research.

### Experimental set-up and design

**Figure 1:**
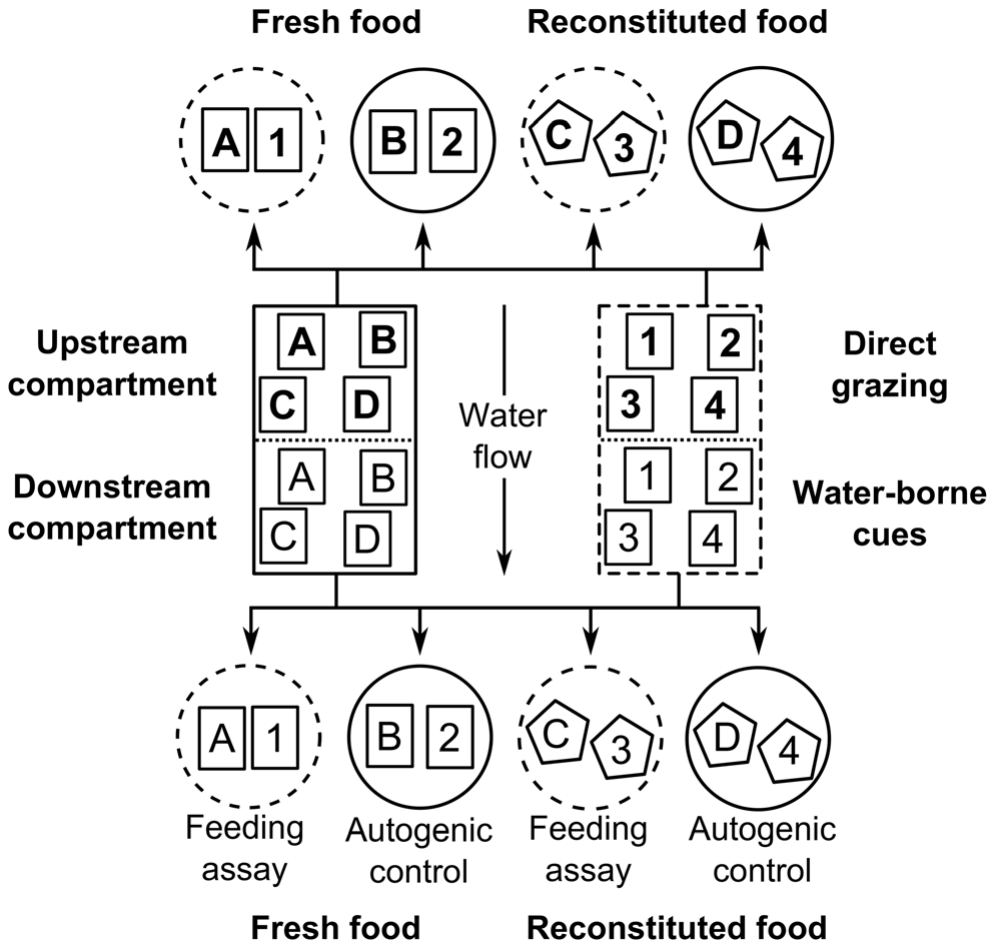
Schematic illustration showing the allocation of * F. vesiculosus* pieces (small rectangles) for a single replicate at one of several sampling time points. Upstream and downstream compartments were separated by a polyethylene mesh and contained 28 pieces each (only 4 shown). From these, 4 pieces were allocated to feeding arenas (circles) at each of the sampling time points. Arrow indicates direction of water flow. Dashed lines indicate containers with herbivores. Solid lines indicate containers without grazers. Letters and numbers indicate control and treated (direct grazing/water-borne cues) pieces of *F. vesiculosus*, respectively. Bold letters and numbers indicate *F. vesiculosus* pieces located in upstream compartments, while all other characters indicate seaweed pieces located in downstream compartments. Pentagons indicate reconstituted food items used for feeding assays with naïve herbivores.

An induction experiment was run in a laboratory of the Biologische Anstalt Helgoland using a flow-through aquaria system in which *F. vesiculosus* was exposed to seawater that previously flowed over grazed conspecifics. Twenty aquaria (25 L volume; 480×230×260 mm) were divided with a polyethylene mesh (mesh size 2 mm) into equally sized upstream and downstream compartments (Fig. 1). Aquaria were irradiated by two fluorescent tubes (Osram Lumilux Daylight L 36W/865) in a 12:12 h light-dark cycle at a mean (± SD) photon flow rate of 125 (±22) µmol m^−2^ s^−1^ (PAR). Filtered North Sea water was pumped over a cotton filter into two 200 L tanks from where aquaria were individually supplied at a mean (± SD) flow rate of 104 (±19) ml/min. Filtered seawater with a mean (± SD) temperature of 19.6±0.7°C flowed through upstream and downstream compartments. To avoid animal escapes from the set-up, each aquarium was covered with a 3 mm thick transparent acrylic plate.

 On 27 July 2012, 8 apical pieces with a mean (± SD) wet mass of 1.77 (±0.44) g lacking visual feeding scars were cut from each of 140 *F. vesiculosus* individuals ( = 1,120 pieces in total). An algal individual was defined as the tissue stemming from a single holdfast. Within 30 minutes all algal pieces were transported to the laboratory, where macroscopic epibionts were gently removed with a soft sponge. To identify genetically identical *F. vesiculosus* pieces in the set-up, seaweed pieces stemming from the same individual were marked with colored threads. Four pieces of each of 7 seaweed individuals were allocated to each compartment of each of 10 treatment and control aquaria ( = 56 pieces per aquarium; 112 per replicate) (Fig. 1). Treatment and corresponding control pieces originated from the same *F. vesiculosus* individual in the field to make sure that potential differences in palatability between both pieces detected in a feeding assay at a specific point in time during the induction phase were not confounded by inter-individual variation in, for example, morphology [25] or chemical composition [26]. Afterwards, seaweed pieces were separately anchored with cable ties (width 1.8 mm) to a polyethylene mesh (mesh size 2 mm) which was placed on the bottom of the aquarium to prevent floating of *F. vesiculosus* pieces.

Two sequential experimental phases were applied: acclimation and induction. During acclimation, algal pieces remained in the set-up for 4 days without grazers to reduce potential effects by cutting the algae as well as putative induced defensive traits, which may have been attained by unknown grazing histories in the field. According to prior studies, 3 days are sufficient to reduce anti-herbivory defenses in *F. vesiculosus* (e.g. [27]). At the end of the acclimation phase, the wet mass of *F. vesiculosus* pieces was determined by blotting them dry with paper towels for 20 seconds and weighing them to the nearest 0.001 g (Sartorius CPA323S, Sartorius, Göttingen, Germany). This was the standard procedure to measure the wet mass of all food items in this study. Afterwards, a feeding assay was started to confirm equal palatability of designated treated and control pieces after acclimation (see details below).

The same day, the induction phase was started by adding 6 *I. baltica* to upstream compartments of all treatment aquaria. Upstream compartments of control aquaria as well as all downstream compartments remained without herbivores. Treatment and control aquaria of each replicate were paired and pairs randomly arranged in the set-up. At the end of the acclimation phase and from day 12 on, 4 genetically identical *F. vesiculosus* pieces of each upstream (direct grazing) and downstream (water-borne cues) compartment were allocated to feeding assays every three days during the 27 day induction phase. The first of these 4 pieces from each compartment was transferred to a feeding arena with naïve grazers. The second piece was allocated to a feeding arena without consumers to determine autogenic wet mass changes during the feeding assay. The third and the fourth piece of each compartment were stored at −80°C and used in feeding assays with artificial food pellets within 4 weeks (Fig. 1).

At the same time, one isopod was removed from the upstream compartment of each treatment aquarium to apply a comparable grazing pressure to remaining seaweed pieces. Furthermore, the wet mass of three *F. vesiculosus* pieces from each upstream compartment was measured at the start of the induction phase and in 3 day intervals thereafter to monitor herbivore consumption during the induction phase. The pieces were chosen randomly for each interval and means were calculated from these pieces as a replicate measure of consumption for statistical analysis (see formula in subchapter ‘feeding assays’).

### Feeding assays

#### Fresh algae

Herbivore preferences for control or treated *F. vesiculosus* were determined in 72 h two-choice feeding assays at several sampling time points during the induction phase. Transparent 8 L plastic aquaria (325×175×185 mm) were used as feeding arenas. Each feeding arena contained naïve herbivores, which were allowed to choose between a *F. vesiculosus* pieces that was either previously grazed or exposed to water-borne cues before and a control piece of genetically identical *F. vesiculosus*. To avoid grazer adaptations, naïve consumers, which were not in contact with *F. vesiculosus* before, were used in feeding assays. One male *I. baltica* was used as consumer in each feeding assay. At the beginning and the end of the feeding assays, the wet mass of the *F. vesiculosus* pieces in the feeding arenas ( = assayed alga) was measured. A second seaweed piece was removed from the same compartment, from where the assayed algae originated and allocated to a feeding arena without grazers to correct herbivore consumption rates for non-feeding related (autogenic) changes in seaweed wet mass. The risk of underestimating error variance and, thus, of committing a type I error was reduced by using the same number of autogenic controls and assayed algae [28]. The following formula was used to calculate the consumption of each assayed *F. vesiculosus* piece (adopted from [29]):
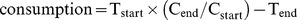
where T_start_ and T_end_ represent the wet mass of an assayed algal piece before and after the feeding assay, respectively, and C_start_ and C_end_ represent the wet mass of the corresponding autogenic control alga and the beginning and the end of the feeding assay, respectively.

#### Reconstituted food

Additional feeding assays with reconstituted food were run to determine whether induced changes in *F. vesiculosus* palatability were due to chemical traits. As described above, the third and the fourth *Fucus vesiculosus* piece, which were removed from each compartment, were stored at −80°C for a period of maximum four weeks prior to the preparation of reconstituted food. Frozen seaweed pieces were lyophilized at −30°C and 0.37 mbar for 24 hours (Christ Beta 1-8 LD plus, Martin Christ Gefriertrocknungsanlagen, Osterode am Harz, Germany). Afterwards, all pieces were ground for 10 seconds at a frequency of 25 Hz with a mixer mill (Schwingmühle MM 400, Retsch Laborgeräte, Haan, Germany). Subsequently, 0.4 g of this powder was mixed with 3.6 ml of molten agar (a blend of 0.02 g agar per one ml of boiling distilled water) after agar had cooled to 45°C to minimize putative thermal destruction of chemical seaweed compounds. This mixture was applied to a polystyrene mosquito mesh (mesh size 1.5 mm) and flattened between two PVC plates coated with wax paper (method adopted from [30]). Uniform thickness of food pellets was guaranteed by placing a 1 mm thick plastic template between both PVC plates. A food item of 2×2 cm area was cut from each pellet after solidification. Each food item was placed in a glass Petri dish (Ø 10 cm, 2 cm height) and transferred to a separate feeding arena. Each feeding arena contained one Petri dish with a food item made from a control *F. vesiculosus* piece and a second Petri dish with a food item made from a *F. vesiculosus* pieces that was either previously grazed or exposed to water-borne cues before. Food items were placed in Petri dishes within feeding arenas to permit correct allocation of fragments that occasionally broke off from food items. Both food items in each feeding arena were weighed and naïve grazers (one male *I. baltica*) were introduced to feeding arenas containing food items made from the third *F. vesiculosus* piece which was removed from each compartment. Autogenic wet mass changes during feeding assays were determined by using reconstituted food pellets made from the fourth *F. vesiculosus* piece which was removed from each compartment (Fig. 1). No grazers were added to feeding arenas that assessed autogenic wet mass changes. Feeding assays were terminated and food items re-weighed after 3 days or when ≥50% of one food item was consumed, whichever came first.

### Statistical analysis

#### Consumption of inducers during induction phase

Consumption of *F. vesiculosus* by *I. baltica* during the induction phases was analyzed by two-tailed paired *t*-tests, comparing consumption between different 3 day intervals of the induction phase.

#### Feeding assays

Herbivore consumption rates from feeding assays performed directly after the acclimation phase were analyzed by one-tailed paired *t*-tests.

Repeated-measures analyses of variances (RM-ANOVAs) were applied to test for the effect of direct grazing by *I. baltica* and isopod grazing on conspecific neighbors on the palatability of *F. vesiculosus* (within-subjects measure: 2 levels, fixed) at different times during the induction phase (between-subjects measures: 6 levels, fixed). RM-ANOVA was used because treatments were not independent and standard ANOVA cannot be properly applied when two food types are simultaneously offered to the same individual consumer [31]. As the within-subject factor had only two levels (control vs. treatment), testing for sphericity is not applicable [32].

Due to ambiguous selection of an appropriate error term for post-hoc tests involving within-subject by between-subject interactions, no post-hoc tests were computed for time×grazing interactions [33]. Instead, one-tailed paired *t*-tests (due to experimental evidence that direct grazing and water-borne cues induce anti-herbivory defenses in *F. vesiculosus*, e.g. [10]) were performed for each time separately. Normal distribution of differences in the consumption of treated and control seaweed pieces was confirmed using the Kolmogorov-Smirnov test.

## Results

### Consumption of inducers during induction phases

**Figure 2:**
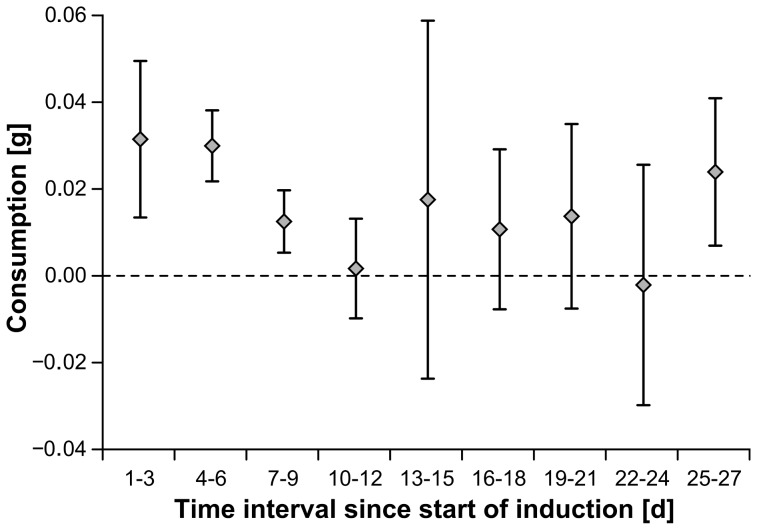
Mean and 95% confidence intervals of *Fucus vesiculosus* consumption by *Idotea baltica* during 3 day intervals in the induction phase (n = 10). Stippled line marks level of no consumption. Confidence intervals overlapping with stippled line indicate intervals when consumption was statistically not significantly different from zero. Different letters indicate significant difference in consumption.

During the induction phase isopods consumed on average 7.7% of initial *F. vesiculosus* wet mass. Inducer consumption varied significantly during the induction phase (Table 1, Fig. 2). With 36 *t*-tests performed in total, the probability of finding 7 tests below p<0.05 by chance is 0.001 [34].

**Table 1:** Results of two-tailed paired *t*-tests comparing isopod consumption between different 3 day intervals of the induction phase (n = 10).

Time interval since start of induction [d]	1–3	4–6	7–9	10–12	13–15	16–18	19–21	22–24	25–27
1–3	-	0.851	0.056	**0.012**	0.559	0.093	0.077	**0.047**	0.505
4–6		-	**<0.001**	**<0.001**	0.536	0.063	0.065	**0.027**	0.501
7–9			-	**0.004**	0.794	0.842	0.903	0.274	0.183
10–12				-	0.467	0.285	0.314	0.763	**0.031**
13–15					-	0.794	0.861	0.429	0.765
16–18						-	0.826	0.404	0.181
19–21							-	0.253	0.476
22–24								-	0.202
25–27									-

Significant p-values, i.e. α≤0.05, in bold.

### Feeding assays

At the end of the acclimation phase, the palatability of *F. vesiculosus* pieces in designated control and treatment aquaria was neither significantly different when tested in assays using fresh algae (one-tailed paired *t*-test: direct grazing *t*_9_ = 0.18, p = 0.429 and water-borne cues *t*_9_ = 0.29, p = 0.388) nor reconstituted food (one-tailed paired *t*-test: direct grazing *t*_9_ = 0.30, p = 0.385 and water-borne cues *t*_9_ = 0.16, p = 0.438).

**Figure 3:**
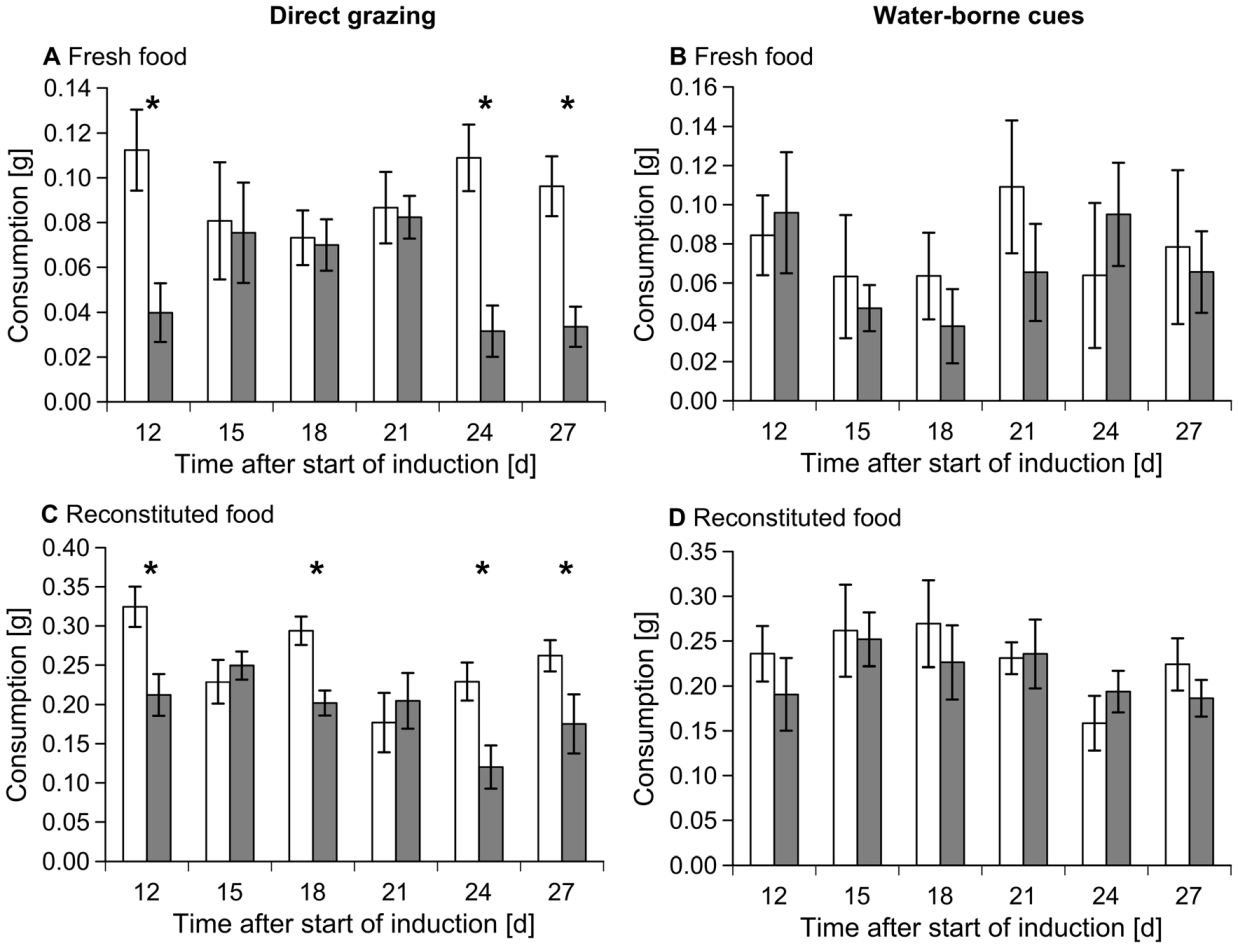
Mean ± SE consumption of *Fucus vesiculosus* by *Idotea baltica* in two-choice feeding assays during the induction phase (n = 10). Isopod consumption of fresh and reconstituted *Fucus vesiculosus* pieces that were previously grazed by *Idotea baltica* (A and C) or located downstream of isopod-grazed conspecifics (B and D) (grey bars) and seaweed pieces which were not exposed to grazing or located downstream of grazed *F. vesiculosus* before (controls; open bars). Asterisks indicate significant results of one-tailed paired *t*-tests comparing distribution of differences between control and grazed pieces against the null-hypothesis of no difference.

RM-ANOVA results did not indicate a significant preference of isopods for fresh and reconstituted *F. vesiculosus* pieces that were located downstream of ungrazed control *F. vesiculosus* pieces compared to seaweed pieces that were located downstream of isopod-grazed conspecifics during the induction phase. There was no significant interaction between treatment and time (Table 2, Fig. 3).

**Table 2:** Results of the 2-factorial RM-ANOVA for the induction phase, comparing feeding rates affected by treatment (control vs. water-borne cues/direct grazing) and time.

	Water-borne cues				Direct grazing			
	df	MS	F	p	df	MS	F	p
Fresh food								
Time	5	0.005	0.72	0.612	5	9.36×10^−4^	0.42	0.830
Error	54	0.007			54	2.21×10^−3^		
Treatment	1	0.003	0.33	0.569	1	0.04	15.85	0.000
Treatment×Time	5	0.004	0.46	0.804	5	0.01	2.53	0.039
Error	54	0.008			54	2.67×10^−3^		
Reconstituted food								
Time	5	0.02	2.16	0.072	5	0.03	3.97	0.004
Error	54	0.01			54	0.01		
Treatment	1	0.01	0.47	0.494	1	0.10	12.17	0.001
Treatment×Time	5	0.01	0.32	0.899	5	0.02	2.49	0.042
Error	54	0.02			54	0.01		

Consumption was assessed in two-choice feeding assays using either fresh or reconstituted *Fucus vesiculosus* (n = 10).

In contrast, isopods significantly preferred ungrazed *F. vesiculosus* to previously grazed seaweed pieces in both fresh and reconstituted food feeding assays. In addition, a significant interaction between isopod grazing and time was found in assays using fresh and reconstituted *F. vesiculosus* pieces (Table 2).

One-tailed paired *t*-tests, that analyzed feeding preferences at each time, revealed that *I. baltica* significantly preferred fresh control *F. vesiculosus* to previously grazed algae pieces 12, 24, and 27 days after the onset of grazing (Table 3, Fig. 3A). Reconstituted food made from previously ungrazed seaweed pieces was preferred to reconstituted food made from previously grazed *F. vesiculosus* 12, 18, 24, and 27 days after the start of the induction phase (Table 3, Fig. 3C).

**Table 3:** Results of one-tailed paired *t*-tests comparing controls and seaweed pieces that were previously exposed to direct grazing by *I. baltica*.

Time [d]	Fresh algae		Reconstituted food	
	*t*	p	*t*	p
0	0.18	0.429	0.30	0.385
12	3.64	**0.003**	3.44	**0.004**
15	0.14	0.447	−0.55	0.495
18	0.18	0.430	3.28	**0.005**
21	0.25	0.403	−0.46	0.456
24	3.74	**0.002**	3.41	**0.004**
27	3.89	**0.002**	1.85	**0.049**

Consumption by conspecific consumers was assessed in feeding assays using either fresh or reconstituted food pieces of *F. vesiculosus* (n = 10). Time = days after start the of the induction phase. Significant p-values, i.e. α≤0.05, in bold.

## Discussion

The induction experiment did not suggest induction of defenses in response to neighbor grazing in *F. vesiculosus*. However, the same experiment clearly showed a significant reduction in palatability of directly grazed upstream seaweed pieces and provided strong evidence for induced defenses in these *F. vesiculosus* pieces. The finding that bioassays conducted with reconstituted food and fresh algae showed very similar results confirms the induction of chemical anti-herbivory traits. Furthermore, *F. vesiculosus* showed a pulsating temporal defense pattern. Because different genetic individuals were used for feeding assays at each sampling time point, the observed temporal pattern may have been caused by genetic differences between *F. vesiculosus* individuals. However, pulsating defenses have been repeatedly shown for *F. vesiculosus* in the recent past [21], [22] and, in addition, were also found in the related seaweed species *Ascophyllum nodosum* [15]. Therefore, it is most likely that short-term variations in seaweed palatability are an intrinsic feature of the *F. vesiculosus* anti-herbivory defense that may entail economic, ecologic and/or evolutionary benefits [21].

The observed lack of a response to water-borne cues released by isopod-grazed neighbors is therefore surprising because fast-swimming isopods were shown to frequently switch between *F. vesiculosus* individuals [35], [36] and may be suggested to imply a relatively high risk of attack for nearby seaweed individuals. Although this result partially coincides with findings reported by Yun and colleagues [8], it deviates from several studies reporting on induction of anti-herbivory defenses in Baltic *F. vesiculosus* located downstream of isopod-grazed conspecifics [10], [13]. Therefore, this study may provide evidence for geographic variation in the ability to emit and/or receive water-borne cues in response to herbivory between genetically distinct *F. vesiculosus* populations originating from Helgoland (North Sea) and the Baltic Sea [37], [38]. Inter-population differences in the ability of seaweeds to induce anti-herbivory defenses were, for instance, reported in European and North American populations of a closely related seaweed (*Ascophyllum nodosum*) [39]. Long and colleagues [40] suggested that this variation may be related to a difference in consumer pressure between European and North American sites. Similarly, *F. vesiculosus* individuals from North Sea and Baltic Sea populations may have evolved different abilities to ‘communicate’ with each other due to either different grazing pressures of *I. baltica* [41] and/or different abiotic conditions between the North and Baltic Seas.

The Baltic Sea is a semi-enclosed virtually tideless habitat [42] where *F. vesiculosus* forms dense and almost mono-specific stands [10]. In such an environment, where water-flow is low and adjacent seaweeds are in very close proximity, the benefits of seaweed-seaweed communication (e.g. increased efficacy of the anti-herbivory defense at the population level [43] or increased inclusive fitness by warning close relatives growing nearby [44]) may outweigh costs affiliated with the production of water-borne cues.

In contrast, the study site on Helgoland represents a relatively exposed habitat with a mean tidal range of about 2.3 m [45] where *F. vesiculosus* forms only moderately dense stands (C. Flöthe, personal observation). In such a high-flow environment where *F. vesiculosus* individuals are as well not in direct contact to each other, water movement may dilute or wash away chemical cues to such an extent that the transmission of these cues between single *F. vesiculosus* individuals is strongly impeded or even becomes impossible. In addition, the release of water-borne cues is suggested to incur a metabolic cost [17] and may cause even higher costs when info-chemical are used by ‘unauthorized’ receivers, such as additional herbivores or unwanted predators [46]. Therefore, it may be assumed that the benefits of seaweed-seaweed ‘communication’ do not outweigh costs affiliated with the production of water-borne cues at the study site. Thus, investing resources in growth and reproduction rather than the synthesis and emission of water-borne cues may offer greater benefits to Helgoland *F. vesiculosus*.

However, straightforward generalizations based on the results obtained in this study should be made carefully. A high degree of variation was shown to exist in the anti-herbivory response of *F. vesiculosus* (e.g. [10]) and ‘communication’ between *F. vesiculosus* individuals in response to another herbivore species at the same site cannot be excluded. Furthermore, even if defense induction in response to water-borne cues released by isopod-grazed conspecifics may not occur in wave-exposed Helgoland *F. vesiculosus*, it may occur at the same place in systems with calm water conditions, such as tide pools [47].
